# Global Tracking in Human Gliomas: A Comparison with Established Tracking Methods

**DOI:** 10.1007/s00062-013-0198-x

**Published:** 2013-01-18

**Authors:** T. Nguyen-Thanh, M. Reisert, C. Anastasopoulos, F. Hamzei, T. Reithmeier, M. S . Vry, V. G. Kiselev, A. Weyerbrock, I. Mader

**Affiliations:** 1 Department of Neuroradiology, University Medical Centre Freiburg, Breisacher St. 64, 79106 Freiburg, Germany; 2 Department of Radiology, Medical Physics, University Medical Centre Freiburg, Freiburg, Germany; 3 Gräfliche Kliniken Moritz Klinik GmbH & Co. KG, Bad Klosterlausnitz, Germany; 4 Department of Neurology, University Medical Centre Freiburg, Freiburg, Germany; 5 Department of Stereotactic and Functional Neurosurgery, University Medical Centre Freiburg, Freiburg, Germany; 6 Department of Neurosurgery, University Medical Centre Freiburg, Freiburg, Germany; 7 Department of Radiology, Hue University College of Medicine and Pharmacy, Hue, Vietnam; 8 Clinic of Neuropediatrics and Muscular Diseases, University Medical Centre Freiburg, Mathildenst. 1, 79106 Freiburg, Germany; 9 Germany Clinic of Neurosurgery, Munich Municipal Hospital Group, Englschalkinger St. 77, 81925 Munich, Germany

**Keywords:** Global tracking, FACT algorithm, Probabilistic tractography, Glioma, Eigenvalues, Global Tracking, FACT Algorithmus, Probabilistische Faserbahndarstellung, Gliome, Eigenwerte

## Abstract

**Purpose:**

Global tracking (GT) is a recently published fibre tractography (FT) method that takes simultaneously all fibres into account during their reconstruction. The purpose of this study was to compare this new method with fibre assignment by continuous tracking (FACT) and probabilistic tractography (PT) for the detection of the corticospinal tract (CST) in patients with gliomas.

**Methods:**

Tractography of the CST was performed in 17 patients with eight low grade and nine anaplastic astrocytomas located in the motor cortex or the corticospinal tract. Diffusions metrics as fractional anisotropy (FA), mean (MD), axial (AD) and radial diffusivity (RD) were obtained. The methods were additionally applied on a physical phantom to assess their accuracy.

**Results:**

PT was successful in all (100 %), GT in 16 (94 %) and FACT in 15 patients (88 %). The case where GT and FACT, both, missed the CST showed the highest AD and RD, whereas the one where FACT algorithm, alone, was not successfully showed the lowest AD and RD of the group. FA was reduced on the pathologic side (FA_path_ 0.35 ± 0.16 (mean ± SD) versus FA_contralateral_ 0.51 ± 0.15, *p*_corr_ < 0.03). RD was increased on the pathologic side (RD_path_ 0.67 ± 0.29 × 10^−^^3^ mm^2^/s versus RD_contralateral_ 0.46 ± 0.08 × 10^−^^3^ mm^2^/s, *p*_corr_ < 0.03). In the phantom measurement, only GT did not detect false positive fibres at fibre crossings.

**Conclusion:**

PT performed well even in areas of increased diffusivities indicating a severe oedema or disintegration of tissue. FACT was also susceptible to a decrease of diffusivities and to a susceptibility artefact, where GT was robust.

## Abstract (de)

**Ziel:**

Global Tracking (GT) ist eine neue Faserbahn-Darstellungsmethode, bei der simultan alle Fasern gleichzeitig in die Faserrekonstruktion mit einbezogen werden. Das Ziel dieser Arbeit war, diese Methode mit dem FACT Algorithmus und der probabilistischen Faserbahndarstellung (PT) anhand der Darstellbarkeit der Pyramidenbahn (CST) bei Gliomen zu vergleichen.

**Methoden:**

Der CST wurde bei 17 Patienten mit acht niedrig-malignen Astrozytomen und neun anaplastischen Astrozytomen im motorischen Kortex oder der motorischen weißen Substanz dargestellt. Diffusionsmaße wie die fraktionelle Anisotropie (FA), die mittlere (MD), radiale (RD) und axiale Diffusivität (AD) wurden bestimmt. Zusätzlich wurden die Traktographie-Methoden an einem physikalischen Phantom getestet, um ihre Genauigkeit abzuschätzen.

**Ergebnisse:**

PT war in allen (100 %), GT in 16 (94 %) und FACT in 15 Fällen (88 %) erfolgreich. Der Fall, bei dem GT und FACT den CST verfehlten, zeigte die höchste AD und RD, während der Fall, bei dem FACT allein nicht erfolgreich war, die niedrigste AD und RD aufwies. Die FA war auf der pathologische Seite signifikant geringer als auf der gesunden Seite (FA_path_ 0,35 ± 0,16 (Mittelwert ± Standardabweichung) versus FA_contralateral_ 0,51 ± 0,15, *p*_corr_ < 0,03). Die RD war auf der pathologischen Seite signifikant erhöht (RD_path_ 0,67 ± 0,29 × 10^−^^3^ mm^2^/s versus RD_contralateral_ 0,46 ± 0,08 × 10^−^^3^ mm^2^/s, *p*_corr_ < 0,03). Bei der Phantommessung wurden lediglich beim GT keine falsch positiven Fasern an Faserkreuzungen gefunden.

**Zusammenfassung:**

PT konnte immer den CST darstellen, selbst bei erhöhten Diffusionsmaßen, die auf ein schweres Ödem oder eine Gewebsdestruktion hinweisen. FACT war zusätzlich empfindlich für erniedrigte Diffusionsmaße und ein Suszeptibilitätsartefakt, wohingegen GT sich hier als robust erwies.

## Introduction

**Fig. 1:**
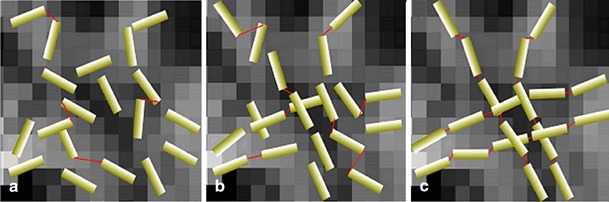
The principle of GT using temperature reduction. The system temperature is reduced from *left* to *right* (**a** to **c**) during the simulated annealing. At high temperature, a diffuse distribution of segments and a low number of connections (*red connecting lines*) exist (**a**). At moderate temperature, a simulated polymerisation starts, which implies an increased number of connections (**b**). At low temperature, aligned and structured segments dominate the configuration, and the crossings are resolved (**c**)

Global tracking (GT) is a new fibre tracking algorithm that considers simultaneously all detectable fibres of the brain. It reconstructs fibres by finding a configuration that describes best the whole set of measured data. The reconstructed fibres are built by small line elements, each of them reflecting a part of the whole diffusion anisotropy [1, 2]. Elements being connected in lines eventually form reconstructed fibres. The process of fibre formation is controlled by a term called temperature. As in a polymerisation process, larger and ordered structures are formed from small elements, when the temperature drops. At high temperature, there is a nearly random distribution of fibres. During a decrease of temperature, the small elements start to connect to each other. At low temperature, the reconstruction volume mainly contains “polymerised” chains, which are aligned and connected, and fibre crossings are resolved, Fig. 1. This method was shown to perform best amongst ten different tractography algorithms on a realistic physical phantom [3].

In contrast to this global method, deterministic fibre assignment by continuous tracking (FACT) [4–6] and probabilistic fibre tractography [7–11] are local methods, where fibres are reconstructed independently from each other, step by step and voxel by voxel. FACT has been shown to be reliable for glioma and cavernoma resection [12–17]. However, FACT can be impaired by low fractional anisotropy (FA) caused by pathologic processes such as oedema or tumour infiltration [18] and by fibre crossings [19].

Probabilistic fibre tractography generates many random tracks by using Monte Carlo simulations and presents results in the form of maps of probability for each voxel to be crossed by a random track [7–11]. Direct pathways between two regions of interest (ROI) can be calculated by combining two maps, the “probabilistic maps of connectivity” [20]. Probabilistic fibre tractography has been applied in optic neuritis [21], multiple sclerosis [22], stroke recovery [23], epilepsy [24] and for surgical planning for deep brain stimulation in lower leg stump pain [25].

The general problem of fibre tractography is that the amount of falsely detected and falsely missed fibres cannot be estimated without histological correlation. Verification in animal models is done for an anatomical description of fibres [26, 27]. A comparison of the depicted fibres with functional data by transcranial magnetic stimulation (TMS) is preferable for a validation of the integrity of the fibres. This has been shown in post-stroke pain for the corticospinal tract (CST) and thalamocortical fibres [28]. In our study, however, seizures were a contraindication for TMS in all patients. To receive at least certain functional information, a clinical scoring of motor impairment, the Fugl-Meyer test (arm section) [29, 30] was performed, serving as a crude, but non-invasive indicator for the integrity of fibres or the motor cortex itself.

The performance of the three tractography methods was further evaluated on a physical phantom with known fibre crossings, fibre bending and fibre splitting [3, 31]. The focus of interest lay in a false positive or false negative detection of fibres. This phantom had been previously used for the competition of ten different tractography methods, of which GT crystallised to perform best [3].

To understand the results of fibre tractography in pathologic conditions, the effects of tumour growth on the tissue such as oedema, compression of fibres or disintegration due to infiltration can be quantitatively expressed by diffusion metrics, for a review see Alexander et al. [32]. The calculation of diffusion metrics is based on the decomposition of the diffusion tensor [33] into three eigenvectors with perpendicular alignment and particular eigenvalues (λ_1_, λ_2_ and λ_3_). The eigenvalue λ_1_ is the largest and is expressed as axial diffusivity (AD). It has been proven to be specific for axonal damage in a mouse model [34, 35]. Radial diffusivity (RD) is the mean of both shorter eigenvalues ((λ_2_ + λ_3_)/2), and was shown to increase in demyelination [35, 36] and to decrease again during remyelination of fibres [34, 36]. Mean diffusivity (MD) is calculated by (λ_1_ + λ_2_ + λ_3_)/3 and is a measure for the magnitude of diffusion. FA relates the sum of the squared differences of the particular eigenvalues minus MD to the sum of the squared eigenvalues:

1 $${\textit{FA}}=\sqrt{3\frac{{{({{\lambda }_{1}}-\textit{MD})}^{2}}+{{({{\lambda}_{2}}-\textit{MD})}^{2}}+{{({{\lambda }_{3}}-\textit{MD})}^{2}}}{2({{\lambda}_{1}}^{2}+{{\lambda }_{2}}^{2}+{{\lambda }_{3}}^{2})}}$$

However, it holds an immanent problem, because several combinations of eigenvalues may result in the same FA value. This is the reason, why AD and RD have come recently more to the fore, when the tissue microstructure has to be assessed [32].

The purpose of this study was to evaluate the new tractography method GT on the corticospinal tract in human gliomas. Here, diffusion is expected to be abnormal, attributed to an altered microstructure of the tissue, e.g. by oedema, infiltration or compression of fibre tracts. It was compared to two established methods (FACT and probabilistic tractography (PT)). To better understand the performance of the algorithms, MD, AD and RD; FA were taken as a mirror of pathologically altered microstructure. As a proof of accuracy, a test was performed on a physical phantom with known diffusion characteristics.

## Materials and Methods

### Patients

Seventeen patients (12 males and 5 females, mean age: 43 years ± 10) with eight low-grade gliomas (WHO grade II) and nine anaplastic gliomas (WHO grade III) participated in this study. The study was approved by the local ethics committee. Written informed consent was obtained from all patients. The patients were prospectively recruited from our outpatient’s department. The tumour had to be located in the pre-central gyrus or in white matter (WM) adjacent to the corticospinal tract. Three patients (# 5, 12 and 15) had a previous stereotactic biopsy 6–10 years before. In these patients, the primary tumour had been located outside of the motor system and had grown into the anatomical areas of interest. The other 14 patients did not have any prior operation. Attributed to the localisation of the tumour in an eloquent brain region (motor system), only a stereotactic biopsy was performed for the determination of the histology. All patients had a history of epilepsy during their course of disease. For details see Table 1.

**Table 1:** Details of findings in tumour anatomy and magnetic resonance imaging (MRI)

Patient	WHO grade	Tumour localisation	Location of interference with CST	Motor fMRI	Susceptibility artefact on T2* EPI images
1	II	L INS, GFM, GFI, GTS, PLIC	WM	M1 intact	No
2	II	R GFS, GPC	GM	Peritumoural activation	2 microbleeds in GFS
3	II	L INS, GTS, PLIC	WM	M1 intact, activation of ipsilateral SMA	No
4	II	L INS, GPC, GSM, GpoC	WM	M1 intact, activation of ipsilateral SMA	No
5	II	L GPC, GFM, SOC	WM	M1 deviated, activation of ipsilateral SMA	1 microbleed in GFM
6	III	R GFI, GR, GTS, GPH, INS, PLIC, PED	WM	M1 intact, activation of ipsilateral SMA	Hemorrhage in tumour
7	III	L GPC, GFM, INS, GpoC, SOC	WM	Activation persists within tumour	Hemorrhage in tumour
8	III	L GFS, GFM, SOC	WM	M1 intact, activation of ipsilateral SMA	Hemorrhage in tumour
9	III	L GFS, GFM, INS, PLIC	WM	M1 intact	2 microbleeds in tumour
10	III	R GpoC, INS, GTS, PLIC, THAL, PED	WM	Peritumoural activation	1 microbleed in tumour
11	III	R GpoC, SOC	WM	M1 deviated, activation of ipsilateral SMA	Hemorrhage in tumour
12	III	R GpoC, CING, SOC	WM	M1 deviated, activation of ipsilateral SMA	1 microbleed in tumour
13	III	L GPC, GFS, GFM, SOC	WM	Peritumoural, contralateral M1 and bilateral SMA activation	Hemorrhage in tumour
14	II	L GPC	GM	M1 deviated, activation of bilateral SMA	1 microbleed in tumour
15	III	L GPC, GFS, Cing	GM	Peritumoural activation, activation of contralateral SMA	No
16	II	L GPC, GFS	GM	Peritumoural activation, activation of ipsilateral SMA	1 microbleed in tumour
17	III	R GpoC, GPC, SOC	WM	Peritumoural, contralateral M1 and ipsilateral SMA activation	No

*CING* cingulate gyrus, *CST* corticospinal tract, *fMRI* functional magnetic resonance imaging, *GFI* inferior frontal gyrus, *GFM* middle frontal gyrus, *GFS* superior frontal gyrus, *GM* gray matter, *GPC* precentral gyrus, *GPH* parahippocampal gyrus, *GpoC* postcentral gyrus, *GR* straight gyrus (rectus), *GSM* supramarginal gyrus, *GTS* superior temporal gyrus, *INS* insula, *M1* primary motor area, *PED* cerebral peduncle, *PLIC* posterior limb of internal capsule, *SMA* supplementary motor area, *SOC* semioval centre, *THAL* thalamus, *WHO* world health organisation, *WM* white matter

### Assessment of Motor Function

The evaluation of motor function was performed at the same day of the magnetic resonance imaging (MRI) acquisition using the Fugl-Meyer test arm section [29, 30]. The following score was used: 0 = no, 1 = slight, 2 = moderate, 3 = marked, 4 = severe motor impairment. Additionally, the point score was included into the statistical evaluation.

### Physical Phantom

All three methods were tested on a physical phantom with known fibre crossings, fibre bending and fibre splitting [3, 31]. We focused on resolution of the following structures: one crossing between two fibre structures of different FA; one sharp fibre bending; one major fibre bundle splitting into three bundles.

### Magnetic Resonance Imaging

To receive seed points for the motor area, functional magnetic resonance imaging (fMRI) was performed at a whole-body 3-T scanner (TIM Trio, Siemens, Erlangen, Germany) by using a 12-channel head coil with a single shot echo planar imaging (EPI) sequence, repetition time (TR) of 2,610 ms, echo time (TE) of 30 ms and voxel size of 3 × 3 × 3 mm^3^. Four blocks (25 s duration) of passive movement of the wrist alternating with five blocks of rest were performed; a pressure-driven arm splintachieved a movement with a frequency of 1 Hz. Diffusion weighted imaging was acquired using a diffusion-sensitive spin-echo EPI sequence with 61 diffusion directions at b = 1,000 s/mm^2^. Parameters were: TR of 10,500 ms, TE of 96 ms and the voxel size was 2 × 2 × 2 mm^3^. During reconstruction, scans were corrected for motion and distortion artefacts based on a reference measurement [37]. A high-resolution T1-weighted anatomical data set (Magnetisation Prepared Rapid Gradient Echo, MPRAGE) was obtained for spatial processing of functional and diffusion tensor imaging (DTI) data. Parameters were: TR of 2,200 ms, inversion time (TI) of 1,100 ms, TE of 2.15 ms, voxel size of 1 × 1 × 1 mm^3^.

### Processing of Data

Processing of functional data was performed using SPM8 (Wellcome Trust Centre for Neuroimaging, University College London, UK). It consisted of motion correction, co-registration of all functional and anatomical images to the diffusion tensor images, smoothing with a Gaussian kernel with full-width at half maximum of 8 × 8 × 8 mm^3^. A *t*-contrast was calculated for “movements” versus “rest”. The seed region in the motor cortex was taken from the global maximum of the statistical *t*-map (*p*_FWE_ < 0.05) and extended by 7 mm in all directions to extend into white matter. The target region was manually placed in the pes pontis, where the corticospinal tract passes through, according to colour maps similar to the method of Kamali et al. [38] who applied this method to somatosensory fibres. Each evaluation was performed in individual space. No normalisation was done, because the tensor calculation was performed on the original images. The T1-weighted images were segmented into grey matter, WM and corticospinal fluid. The WM segment was taken as mask for tractography to include only WM into the analysis.

The calculation of the orientation distribution function (ODF) on high angular resolution diffusion imaging (HARDI) data was performed by in-house software using the constant solid angle approach [39]. Fibre tractography and the calculation of the diffusion metrics were performed by publicly available software (www.uniklinik-freiburg.de/mr/live/arbeitsgruppen/diffusion/fibertools_en.html). To achieve comparable results, the stop criterion for FACT and probabilistic tracking was set to FA = 0.1 to match the effective internal threshold in GT. To avoid any effects of a FA threshold at all, for FACT and probabilistic tracking, also fibre tracts without FA threshold were calculated resulting in identical results. Diffusion metrics such as FA, MD, AD and RD were calculated from a standard rank-2 tensor.

For FACT, the allowed maximal curvature between two consecutive steps was set at 90°. This extremely liberal curvature was chosen to allow the detection of strong fibre bending. The results were displayed as a projection of fibres on anatomical images.

For probabilistic maps of connectivity, visiting maps were calculated by a tracking algorithm that also accounts for the smaller elements of the diffusion distribution by an oscillating largest vector. The number of visits and the direction of entrance were counted and noted. Connecting maps were created by a combination of two maps from each seed point with an entrance into the voxel from opposite sides [20]. To get comparable inter-individual results an intrinsic threshold was set, where the least fibres appeared. The depicted fibres were displayed in three orthogonal sections.

The parameters of GT were chosen as suggested by Reisert et al. [2]. The model parameters were: cylinder’s width 1 mm and length 3 mm, which is reasonable in comparison to a resolution 2 × 2 × 2 mm^3^ for the diffusion tensor imaging (DTI) data. The weight of a cylinder was adapted to the signal statistics (1/4th of brain-averaged anisotropic signal component) [2], which corresponds to a rather dense reconstruction, that is, the number of cylinders per voxel is in the range of 30. Note that the weight parameter behaves similar to a FA threshold. For higher weights the number of fibres is reduced and only regions with a highly anisotropic diffusion distribution show a significant amount of fibres. A lower weight parameter leads to high number of fibres, even in regions with low anisotropy. As the chosen weight parameter is rather low, a relatively high number of 3 × 10^8^ iterations were chosen. The temperature schedule of the cooling phase was chosen exponentially with a starting temperature of 0.1 to a stop temperature of 0.001. The results were displayed as a projection of fibres.

### Image Analysis

The detection of the CST was simply assessed as “found” or “not found”. The diffusion metrics including FA, MD, AD and RD were calculated within the depicted CST (obtained as region of interest from probabilistic maps of connectivity) and plotted along the *z*-axis by using in-house software implemented in Matlab (Version R2009b, MathWorks, Ismaning, Germany). The CST between *z*-coordinates 70 and 10 mm was covered from the semioval centre to the cerebral peduncle. At the point of strongest deviation of the CST caused by mass effect of the tumour, FA, MD, AD and RD of the pathologic and corresponding healthy side were obtained.

For an assessment of deviation of the CST by the tumour mass, an index of deviation (ID) was calculated. At the level of the strongest deviation of the CST, the distance of the middle of the CST on probabilistic maps of connectivity to the midline was obtained for the healthy (D_h_) and the pathological hemisphere (D_p_). Both distances were set into relation by

2 $$\text{ID}=\left| \left( {{\text{D}}_{\text{h}}}-{{\text{D}}_{\text{p}}} \right) \right|/\left({{\text{D}}_{\text{h}}}+{{\text{D}}_{\text{p}}}\right)$$

At the level of the strongest deviation of the CST, a planar area of tumour size was obtained on T2w images by using the area formula of an ellipse. Additionally, b0 images were analysed in all the patients to assess microbleeds and hemorrhages.

Motor activation was visually assessed as “M1 unaffected by tumour”, “M1 deviated but unaffected”, “peritumoural activation”, “activation of the SMA” and “contralateral activation”.

### Statistics

Statistics were calculated by using IBM^®^ SPSS^®^ Statistics 19 (SPSS Inc., Chicago, Illinois, USA). A Pearson correlation coefficient was calculated to test the correlation between the grade of motor impairment (Fugl-Meyer test), the diffusion metrics and the ID. After proven normal distribution, a paired *t*-test was performed between the diffusion metrics of the healthy and pathological side. A Bonferroni correction for multiple testing was applied.

## Results

### Motor Function

According to the arm section of the Fugl-Meyer test, ten patients had no (grade 0), two had slight (grade 1), one had moderate (grade 2) and four had marked (grade 3) motor impairment, Table 2. There was no difference between low grade and high-grade gliomas. Only in a subgroup with tumour localisation merely in WM (patients #1, 3–13 and 17) there was a weak correlation between AD and the Fugl-Meyer test score only at an uncorrected *p*-value, *r* = 0.64, *p*_uncorr_ < 0.03. No correlation could be found between the Fugl-Meyer test score and the ID, *r* = 0.16, *p*_uncorr_ = 0.9.

**Table 2:** Details of tumour size, deviation of CST and diffusion metrics

Patient	WHO grade	Classification of motor impairment	Fugl-Meyer test point score	Planar area of largest tumour mass	ID of the CST	FA	MD	AD	RD
1	II	1	63	49	0.23	0.3	0.69	0.9	0.59
2	II	0	66	9	0.14	0.3	1.04	1.35	0.89
3	II	1	64	10	0.08	0.3	0.76	0.98	0.65
4	II	0	66	17	0.16	0.8	0.67	1.43	0.29
5	II	0	66	20	0.07	0.5	0.65	1.05	0.45
6	III	3	50	31	0.14	0.3	0.35	0.64	0.21
7	III	3	45	28	0.23	0.2	1.33	1.58	1.2
8	III	0	66	45	0.13	0.3	0.72	0.92	0.62
9	III	3	51	27	0.06	0.4	0.64	0.88	0.48
10	III	2	58	62	0.38	0.4	0.72	1.08	0.53
11	III	0	66	19	0.11	0.6	0.68	1.14	0.44
12	III	0	66	30	0.11	0.2	0.79	0.98	0.69
13	III	3	40	19	0.00	0.2	1.32	1.56	1.2
14	II	0	66	6	0.09	0.3	0.86	1.25	0.67
15	III	0	66	7	0.09	0.3	1.13	1.48	0.95
16	II	0	66	8	0.11	0.2	0.89	1.1	0.79
17	III	0	66	7	0.05	0.4	1.04	1.48	0.82
Mean from ipsilateral side ± SD				23.2 ± 16.4	0.13 ± 0.8	0.35* ± 0.16	0.84 ± 0.26	1.16 ± 0.28	0.67* ± 0.29
Mean from contralateral side ± SD						0.51 ± 0.15	0.67 ± 0.03	1.08 ± 0.16	0.46 ± 0.08

Classification of motor impairment ranges from “0” = no to “4” = severe motor impairment

Diffusivities are given in units of n ´ 10^−3^ (mm^2^/s)

Area of tumour is given in (mm^2^)

*FA* fractional anisotropy, *ID* index of deviation, *MD* mean diffusivity, *AD* axial diffusivity, *RD* radial diffusivity, *SD* standard deviation

^*^ *p*_corr_ < 0.03

Cortical activation was distributed around the tumour in six patients (#2, 10, 13, 15, 16 and 17). Activation within tumour was found in one patient (#7), and of the contralateral primary motor cortex in another patient (#13). Activation of ipsi- or contralateral supplementary motor area (SMA) was present in ten patients (#3–6, 8, 11, 12, 15, 16 and 17) and of bilateral SMA in two patients (#13 and 14). The depicted motor activation resembled a previously described pattern of motor activation in brain tumours [40], Table 1.

### Fibre Tractography

**Fig. 2:**
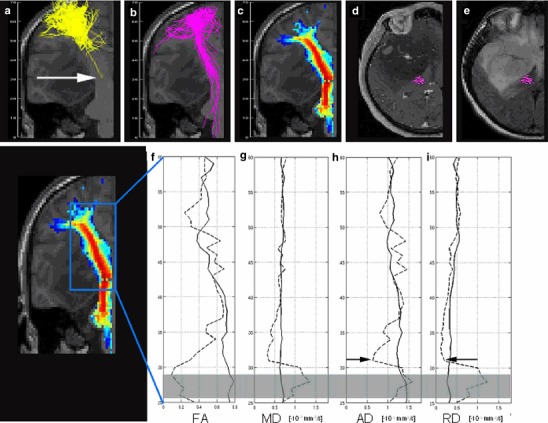
Patient #6 with a *right* fronto-temporal glioma with marked motor impairment. FACT algorithm (**a**) failed in the depiction of the CST, whereas GT (**b**) and probabilistic maps of connectivity (**c**) performed well. Fibres of FACT and GT are given as projections, probabilistic maps of connectivity as cross section. The diagrams display the values of *FA* fractional anisotropy (**f**), *MD* mean (**g**), *AD* axial (**h**) and *RD* radial diffusivity (**i**) (given in *n* × 10^−3^ mm^2^/s), obtained from the CST on the pathologic (*dotted line*) and the contralateral side (*solid line*) from the *top* (*z* = 60 mm) to the *bottom* (*z* = 25 mm). All diffusivities were restricted at the level of *z* = 32 mm (*arrows* in **a**, **h** and **i**). Transversal contrast enhanced T1-weighted (**d**) and T2- weighted images (**e**) at this level showed a mass effect, dislocation and compression of the CST. The *grey bar* in **f** through **i** indicates an area of distortions in the diffusion weighted images in this case

**Fig. 3:**
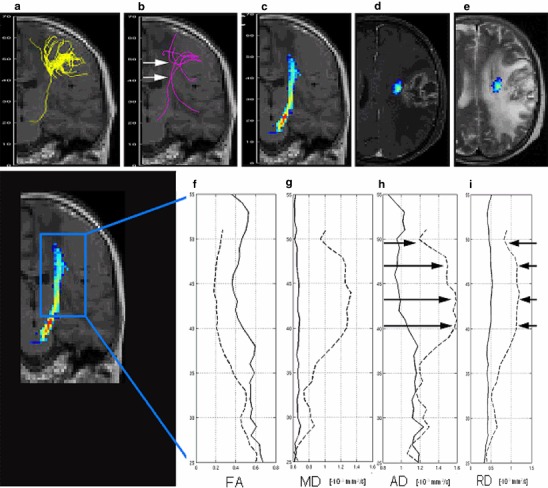
Patient #7 with a *left* frontal glioma with marked motor impairment. FACT algorithm (**a**) and GT (**b**) failed in the depiction of the CST, whereas probabilistic maps of connectivity (**c**) could depict it. Fibres of FACT and GT are given as projections, probabilistic maps of connectivity as cross section. Plots display the values of *FA* fractional anisotropy (**f**), *MD* mean (**g**), *AD* axial (**h**) and *RD* radial diffusivity (**i**) (given in *n* × 10^−3^ mm^2^/s), obtained from the CST on the pathologic (*dotted line*) and contralateral side (*solid line*) from the *top* (*z* = 55 mm) to the *bottom* (*z* = 25 mm). All diffusivities were increased at the level between *z* = 40–48 mm (*arrows* in **b**, **h** and **i**). Transversal contrast enhanced T1-weighted (**d**) and T2-weighted images (**e**) at this level showed a contrast-enhancing tumour in the direct vicinity of the CST and a high signal on T2-weighted images. Both, the tumour infiltration and the vasogenic oedema cannot be distinguished from each other

On visual inspection, the CST was shown with the same anatomical course in each particular patient by all three tracking methods, except in two cases. FACT algorithm of the CST was not successfully in patients #6 and #7, Figs. 2 and 3. Both cases were WHO grade III and suffered from a marked motor impairment. GT succeeded to reconstruct the CST in 16 cases. It failed in one case (patient #7), where FACT algorithm was also unsuccessful, Fig. 3. Probabilistic maps of connectivity of the CST could be obtained in all cases, even in those with large tumours and mass effect. The ID of the CST correlated with the planar tumour size at the height of the strongest deviation, *r* = 0.72, *p*_corr_ < 0.01. The mean ID ± SD was 0.13 ± 0.08, the mean planar tumour size ± SD was 23.2 ± 16.4 mm^2^.

### Changes of Diffusion Metrics

**Fig. 4:**
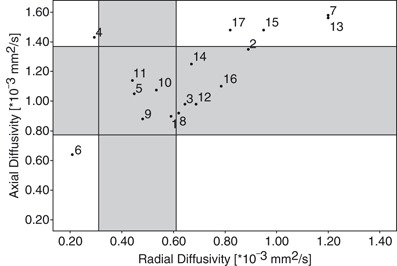
Lambda chart of RD and AD. The *scatter diagram* displays the individual relation between RD and AD diffusivity for each patient. The *small circles* represent the values (given in *n* × 10^−3^ mm^2^/s), the numbers indicate the patient’s number. The *grey horizontal* and *vertical bars* indicate the mean ± 2SD for RD and AD obtained from the contralateral side. Patients #5, #1, 5, 9, 10 and 11 were in the normal range, whereas patient #6 was at one end of the extreme. Patients #7and #13 were on the other. Both (patients #7and #13) showed a contrast enhancement on T1-weighted images. FACT failed in the detection of the CST in patient #6, where the CST was severely compressed. FACT and GT could not display the CST in patient #7, where vasogenic oedema and disruption of the CST by contrast-enhancing tumour were indistinguishable. In patient #13, CST was depicted by all methods, although RD and AD were similar to patient #7. Contrast enhancement, however, was more distant from the CST, see Fig. 6

FA was reduced on the pathologic side compared to the contralateral side: FA_path_ 0.35 ± 0.16 (mean ± SD) versus FA_contralateral_ 0.51 ± 0.15, *p*_corr_ < 0.03. RD was increased on the pathologic side RD_path_ 0.67 ± 0.29 × 10^−3^ mm^2^/s versus RD_contralateral_ 0.46 ± 0.08 × 10^−3^ mm^2^/s, *p*_corr_ < 0.03). MD and AD did not show significant differences between healthy and pathologic sides. Particular values are given in Table 2. The absolute values of the diffusion metrics from the contralateral side were in accordance to the literature [32, 41]. A lambda chart [42] was created to visually represent changes of AD and RD of the particular cases, Fig. 4. The mean ± 2 SDs from the contralateral side were indicated for comparison. Ten patients had an increased and two patients had a reduced RD. Five patients had an increased and two patients had a reduced AD. There was no significant difference between WHO grade II and III tumours concerning FA, MD, AD and RD.

### Phantom Measurement

**Fig. 5:**
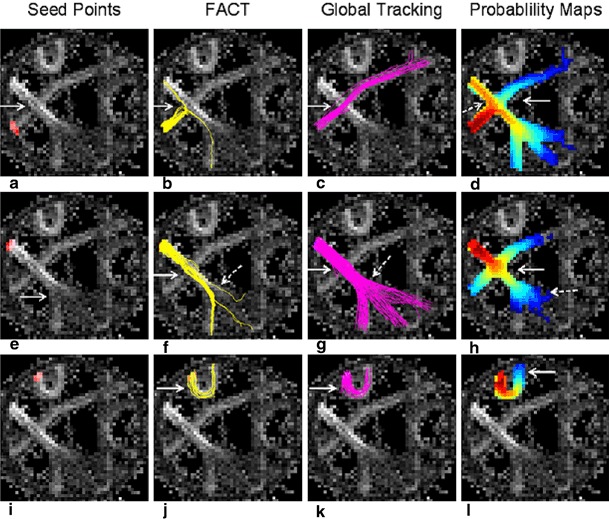
Comparison of the tractography algorithms on the physical phantom. The phantom (3) is displayed as FA map. It contains a fibre crossing on the *left* side (*white arrow* in **a**). The tract crossing from the *left upper* corner to the *right lower* direction (*seed point* indicated in *red* on **e**) has a higher FA than its counterpart from the *left lower* area to the *right upper* direction (*seed point* on **a**). In the upper part of the phantom a strong fibre bending is present (*seed point* on **i**). In the *lower* part, a fibre splitting is contained (*white arrow* in **e**). FACT was not able to overcome the crossing over the tract with the higher FA (*arrow* in **b**). Instead, false fibres were found. Along the tract with the higher FA, FACT successfully overcame the crossing (*solid arrow* in **f**). It only found minor and unequal parts after fibre splitting (*dotted arrow* in **f**). The sharp bending tract was well found (*arrow* in **j**). GT overcame the fibre crossing from both sides (*solid arrow* in **c** and **g**), was able to detect fibres after splitting in nearly equal portions (*dotted arrow* in **g**) and went successfully along the fibre bending (*arrow* in **c**). PT was only partially successful in passing the crossing (*solid arrows* in **d** and **h**), but found false positive fibres during the crossing of the tract with higher FA (*dotted arrow* in **d**) and during the crossing along the tract with higher FA (*solid arrow* in **h**). The fibre splitting was not sufficiently depicted (*dotted arrow* in **h**). PT went successfully along the fibre bending (*solid arrow* in **l**)

The phantom measurement showed that FACT algorithm was not able to overcome one crossing, and resulted in the detection of false fibres. The fibre bending was well found, but of three splitting fibres only one was detected. GT successfully overcame the crossings, passed along the bending and depicted all three splitting bundles. Probabilistic maps of connectivity passed the crossing, but found false positive fibres. Fibre splitting was not sufficiently depicted. It went successfully along the fibre bending, Fig. 5.

## Discussion

A new tractography method has to be evaluated in clinical cases. The most challenging cases for tractography are those, where diffusion metrics such as FA, MD, AD and RD are altered due to changes of the underlying microstructure. In gliomas, changes of the above mentioned diffusion metrics are expected due to oedema, tumour infiltration and compression of fibres by mass effect. For an evaluation of methods, a fibre structure should be chosen that has only few crossings and consists of uniform fibre populations like the pyramidal tract [32]. Therefore, the corticospinal tract in patients with gliomas seemed to be suitable for a comparison of GT with other established methods.

### How did the Tractography Algorithms Perform?

Probability maps of connectivity were not hindered by any changes of the microstructure or mass effect. GT was only hindered in one case (patient #7), which will be discussed along with FACT algorithm. FACT algorithm performed worst with a missing detection of the CST in patients #6 and #7. In both patients, the planar tumour size was above the mean tumour size with 31 mm^2^ (patient #6) and 28 mm^2^ (patient #7) versus a mean tumour size of 23.2 ± 16.4 mm^2^ and a maximal tumour size of 62 mm^2^ (patient #10). This indicates that the tumour size itself is not a crucial factor for the success of a fibre tracking method. The ID was 0.14 for patient #6 and 0.23 for patient #7, compared to a mean ID of 0.13 ± 0.08 and a maximal index of 0.38 in patient #10. The ID is indeed in correlation with the planar tumour size (*r* = 0.72, *p*_corr_ = 0.006), but cannot explain the special cases #6 and #7. Both have an increased ID, but do not reach maximal values like in patient #10, where all three algorithms worked well. The amount of haemorrhage within the tumours and microbleeds in the vicinity of the tumours (Table 1) could also not be identified as influential factor for the success of fibre tracking. Even if patients #6 and #7 did not stand out in the tumour size and ID, they showed the most prominent changes on the lambda chart, Fig. 4. Patient #6 had a maximally reduced AD and RD, whereas patient #7 had a maximally increased AD and RD.

So it is necessary to look at the specific effects of altered microstructure on the success of the fibre tractography methods. Therefore, diffusion metrics reflecting those changes of the microstructure will be discussed.

### Increased Radial Diffusivity

**Fig. 6:**
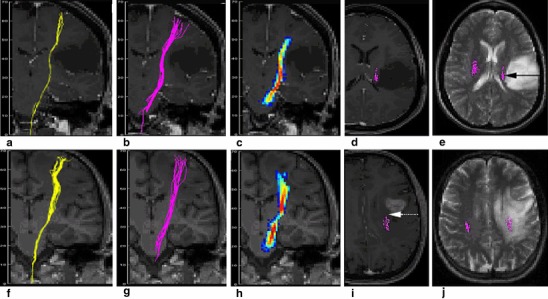
Patterns indicating a different microstructure. All tractography algorithms depicted the CST in patient #4 with a *left* frontal low grade glioma (**a**–**e**), and in patient # 13 with a *left* frontal anaplastic astrocytoma (**f**–**j**). FACT algorithm is displayed in **a** and **f**, probabilistic maps of connectivity in **c** and **h**, and GT in **b**, **d**–**e** and **g**, **i**–**j**. In patient #4, there was a slight compression of the CST, which was surrounded by normal appearing *white* matter (*solid arrow* in **e**). The compression was reflected by a reduced RD, Fig. 4. In patient #13, tractography of the CST successfully depicted the CST, although it ran through an area of *white* matter hyperintensity. Although RD and AD were elevated as in patient #7, the CST was successfully found. This is most likely attributed to the distance between the CST and the contrast-enhancing tumour (*dotted arrow*)

There were ten patients with increased RD in the corticospinal tract. Two cases with the most prominent increase (patients #7 and #13) also had the most prominent increase in AD. In clinical imaging, a large contrast-enhancing tumour with mass effect and a large hyperintensity on T2-weighted images was found in both cases, Fig. 3 (patient #7) and Fig. 6 (patient #13). Both tumours were WHO grade III and lead to a severely impaired motor function. Contrast enhancement indicated a dysfunction of the capillary endothelium leading to capillary leakage and vasogenic extracellular oedema [42], which could explain the increase of RD and AD. On the other hand, it is known that tumour infiltration can extend at least 11 mm beyond the contrast-enhancing rim of a tumour [43] and cannot be distinguished from “pure” vasogenic oedema [42]. In patient #7, the CST in PT was in the direct vicinity of the contrast enhancement, whereas in patient #13 the distance was 22 mm. So the missing detection of the CST by FACT and GT in patient #7 seems to depend on the proximity to the contrast-enhancing tumour.

From the other eight cases with increased RD (patients #2, 3, 8, 12 and 14–17), none of them showed any contrast enhancement in clinical imaging. Only minor mass effect was present which is reflected in no or only slightly impaired motor function. Yuan et al. [41] found an increased RD and MD in ipsilateral normal appearing WM compared to the contralateral side in paediatric low grade tumours (WHO I and II), whereas AD and FA did not reach significance. In a mouse model, an increased RD was found to be accompanied by a lack of myelin and normalised during remyelination in the presence of preserved axons [34–36].

### Reduced Axial Diffusivity

A reduced AD has been described in axonal pathology attributed to ischemia [44], or Waller degeneration after ischemia [45] in the mouse model. AD of peritumoural oedema has been shown to be reduced in an example of a glioblastoma compared to contralateral WM [42], but has not been interpreted so far. Although there are several models about diffusion metrics and gliomas [42, 46, 47], a reduced AD has not been addressed. A compression of bovine cartilage, however, was shown to lead to a decrease of the maximum (λ_1_ or AD, author’s note) and mean eigenvalue [48]. In patient #6, AD and RD were markedly decreased. On clinical images, the CST was strongly compressed at the level of the midbrain, Fig. 2. Whether a compression of CST or a susceptibility artefact arising from the sphenoid cave two slices below the maximal changes of diffusion metrics are responsible for the missing success of the FACT algorithm, cannot be distinguished.

### Reduced Radial and Increased Axial Diffusivity

Patient #4 was also at an extreme end of the lambda chart, Fig. 4. A reduction of RD was accompanied by a slight increase of AD, whereas FA was strongly increased (highest FA value of all data). Clinical imaging showed the tumour core area in the motor cortex lateral to the hand knob. The CST was not directly involved by this WHO II tumour which is reflected by a normal motor function. A contrast enhancement was absent. But a faint mass effect on the CST was visible, see Fig. 6.

All the other less pronounced changes of the diffusion metrics were not accompanied by problems in the detection of the CST indicating a certain robustness of all methods. The newer methods such as probabilistic maps of connectivity and GT, however, seemed to be more robust against reduced AD and RD than FACT. The correlation of the ID with the tumour size points to an anatomically reasonable depiction of the fibres.

### Comparability of the Fibre Tractography Algorithms

Different parameters are important for the success of the particular fibre tractography methods. It is difficult to methodologically compare these methods. For FACT and probabilistic maps of connectivity a FA threshold of > 0.1 was chosen to match an internal threshold of GT that acts like an FA threshold. For GT there is no direct FA-threshold, but the weight parameter (see processing of data) shows a very similar behaviour. It was chosen such that spurious fibres appear only in regions with FA lower than 0.1. To proof whether FACT and probabilistic maps would have performed better without FA threshold, both methods were also calculated without a FA threshold resulting in no changes. In Table 2 it is visible that the FA never dropped below 0.2. The immanent parameters of the different fibre tractography methods are not directly comparable, but they were chosen as described in the literature for probabilistic maps of connectivity [1, 49] and for GT [2], where they had been optimised to a certain extent.

### Proof of Accuracy on a Physical Phantom

As a proof of the accuracy of all three methods concerning the detection of fibres, a test on a physical phantom with known diffusion characteristics [3, 31] was performed, Fig. 5. GT performed best. It did not indicate false positive fibres, resolved crossings and detected fibre splitting. FACT and probabilistic maps of connectivity found false positive fibres. For FACT, one reason might be that the allowed curvature of 90° was too liberal, but on the other hand, with a reduced curvature the strong bending would not have been found. Fibre crossing is a well-known problem of FACT [3, 19]. The detection of fibre splitting by FACT algorithm, however, has only been addressed by Fillard et al. [3]. In their phantom measurement, those methods that used algorithms similar to FACT did not correctly depict the splitting of the fibres. The problems of false positive fibres at fibre crossings and incomplete depiction after fibre splitting in probabilistic tractography has also been addressed by Fillard et al. [3] in the supplementary material section. Our results were nearly identical to their results obtained by the method of Behrens et al. [8]. The phantom, however, does not exactly mirror the microstructure of the brain, so these results have to be considered with caution. But in the presence of only few verification methods of clinical fibre tractography methods, the phantom measurement was helpful, especially as the clinical correlation with the Fugl-Meyer test was not ground-breaking.

## Limitations

A limitation of the study is choosing of a binary classification for the assessment of the CST within the fibre tracking. The aim of the study, however, was to avoid a subjective visual ranking which would have been necessary. Measurements of the thickness of the depicted CST, even in comparison to the contralateral side, seemed to be too dependent on the particular tracking algorithms. Instead, we additionally investigated the microstructure of the tissue mirrored by the diffusion metrics such as AD and RD to understand the limits of each fibre tracking algorithm. It seems to be a limitation that only tumours of grade II and III had been included into the study. The primary aim, however, was to receive a quite homogenous group of pathologies and of involved neurological systems to exclude additional influencing factors from histology (e.g. angioneogenesis or non-brain tissue in metastases). This is also the reason for the low number of patients, because the restricted inclusion criteria of only gliomas of grade II and III, predominantly in the motor system and without prior operation limited the recruiting of patients. Another limitation consists in the fact that no TMS has been performed to assess the functionality of the CST. As all patients suffered from seizures, and as epilepsy is a contraindication for TMS, this method was not performed. Instead, the Fugl-Meyer test (arm section) was included. However, there was only a weak correlation between AD and the Fugl-Meyer test score only at an uncorrected *p*-value, *r* = 0.64, *p*_uncorr_ < 0.03. Moreover, a correlation between the Fugl-Meyer test score was neither present for the ID nor for the tumour size, respectively. The motor function is dependent on multiple factors including cortical involvement by the tumour and the peritumoural reorganisation [40] as well as fibre deviation or fibre disruption. An intra-operative mapping of the CST would also have been desirable. The patients, however, consisted of a cohort who primarily received a stereotactic biopsy. This subgroup had been chosen to avoid any effects of prior surgery onto the fibre tracking. As a small part of proof, the correlation between the ID and the tumour size (*r* = 0.72, *p*_corr_ < 0.01) indicates that at least an anatomical displacement by the tumour can be detected by fibre tracking.

## Conclusion

FACT, probabilistic maps of connectivity and GT are clinically robust methods. FACT was more susceptible to changes of RD and AD and susceptibility artefacts than the others and did not perform in extreme conditions. Probabilistic tractography showed false positive fibresin the phantom measurement. GT performed best in the phantom measurements and showed results which appeared clinically meaningful.

### Acknowledgments

T. Nguyen-Thanh, A. Weyerbrock and I. Mader were supported by the Comprehensive Cancer Centre Freiburg (CCCF), Germany (Seeding Grant 1027013110). T. Nguyen-Thanh, V.G. Kiselev and I. Mader were supported by the German Research Council (DFG: KI 1089/3-1, MA 2343/4-1). The authors thank Mr. Hansjörg Mast for his support.

### Conflict of Interest

The authors declare that there is no actual or potential conflict of interest in relation to this article.
